# Royal Hospital for Diseases of the Chest

**Published:** 1889-10-12

**Authors:** 


### Royal Hospital for Diseases of the Chest.

IS OUR HOSPITAL SYSTEM IN DANGER?

On Monday evening a crowded meeting of the governors of the Royal Hospital for Diseases of the Chest, consisting largely of working men, was held at the institution, in City-road, to consider the questions of (*a*) selling out funded property to meet the liabilities of the hospital, and (*b*) the relations between the medical staff and the late secretary. The meeting, which was presided over by Lord Charles Bruce (the president), lasted nearly three hours, the proceedings being of a very animated character.

The Chairman said he fully realised the critical period in the history of the hospital at which they had arrived, and the very great responsibility of his position. It was not too much to say that upon the result of that meeting, the future of the institution would depend. He hoped the proceedings would be marked by the moderation, dignity, and impartiality which the gravity of the occasion required (hear, hear). It was proposed to sell out £6,000 from the funded property in order to meet the accounts due to the builders, and also to repay the loan to their bankers, who had been very considerate.

Mr. Hope Morley proposed, Mr. Gosnell seconded, and it was resolved almost unanimously to sell out of the funded property a sum not exceeding £6,000 to pay the accounts of the builder and banker.

The Chairman then called upon Mr. Sandeman to read a document, which set forth at length that the conduct pursued by the secretary (Mr. Austin) had interfered with the business of the hospital. Mr. Austin, it appeared, had cast reflections upon the action of the medical staff, and had declared that there was an increasing tendency to make the hospital less an institution for the treatment of patients than a school for the education of the doctors. He had further declared that many patients recommended by subscribers were refused admission to the wards, and that only such cases were specially treated as were likely to prove of scientific value to the medical staff. One of these cases was that of a poor girl upon whom the operation of tracheotomy was performed, and another was that of a man upon whom the experiment, as he called it, of introducing thirty-two feet of steel wire into his body was tried. The patient died. In the meantime a letter was addressed by the honorary medical staff to the chairman of the council, asking that the statements should be investigated, and tendering their resignation. This letter was signed by Dr. Hensley, the senior physician; Dr. T. Gilbart Smith, physician; Mr. Pearce Gould, surgeon, and the whole of the acting medical staff. Mr. Jonathan Hutchinson, consulting surgeon to the hospital, also wrote, fully approving the action taken by his colleagues. The next letter, addressed to the chairman, was from Dr. Hensley, who defended the staff's action with regard to the two cases referred to. The first was that of Edith Jacob, who rapidly developed dangerous symptoms. This was an emergency case, and in performing the operation of tracheotomy there was a reasonable hope of saving the child's life. The operation was remarkably well done by the house physician under his direction, and was completely successful. Bronchitis, however, supervened, and the child sank from exhaustion. The post-mortem examination proved that the operation was the best thing that could have been done under the circumstances. The next case was that of John Raper. He was in a condition of extreme danger, and Dr. White at once advised his admission. By his own account this patient was apparently quite well, but he was afflicted with an aneurism. The object of the operation was to strengthen the wall of the aneurism in the same way that the dam of a reservoir might be strengthened, by damming up the weak part. It was necessary that a considerable length of wire should be introduced. The operation was performed by Mr. Pearce Gould, in the presence of the physicians, and the case was fully discussed at a meeting of the Medico Chirurgical Society, and was published in its *Transactions* some years ago (1885). As a result of their investigations the council came to the conclusion that the charges brought by the secretary against the medical staff were without foundation, and they called upon Mr. Austin to resign his office. At the same time the medical staff were requested to withdraw their resignations. This they eventually did, and Mr. Austin's (the secretary) resignation was subsequently accepted by the council.

The governors having listened to this explanation, proceeded to offer various suggestions, and the meeting soon became unruly. One member proposed to read extracts from a writer who was directly at variance with Dr. Hensley on the subject of the operation on Raper, but Dr. Tidy energetically protested against the injustice of this course, declaring that extracts read from a medical report would probably convey a wrong impression. In this view he was supported by the chairman.

Mr. Henry Maudsley proposed: "That this meeting, having heard the statement made by the council, hereby endorses its action, and expresses full confidence in the council and in the medical staff."

Mr. Horran seconded the motion.

Mr. Austin, the late secretary, being called upon, said: Whilst he made no charge against the medical staff, he was convinced that there was a tendency to make the hospital an institution rather for the doctors than for the patients. In support of that contention he was about to quote from an anonymous publication ("Dying Scientifically,"* by Æsculapius Scalpel.); but as there were requests that the name of the author should be given, the chairman stated that it was only fair that as the charges had been made against the medical staff, whose names were known, the writer of the publication from which Mr. Austin desired to quote should be given.

Mr. Austin replied that as the name of the author was given to him in confidence (voices, "It is no secret") he declined to mention it, and therefore must forbear from reading the extracts he desired.

Dr. Gilbart Smith then interposed, and said he happened to know the author, who was a recent student at a hospital school, and of no importance as an expert.

The Rev. W. Devereux, vicar of St. Mary's, Hoxton, regretted that the hospital authorities could not settle the dispute in private. Their secretary appeared to have been dismissed in a most summary way, although he had worked very hard for the hospital. In fact, no man had done so much for it as he had done. His own excellent private character had also brought many persons to take an active interest in and to support the charity. It was sincerely to be hoped that so worthy a man would be retained as an official. As governors they were entitled to know what was going on in the hospital, because he and others sent a large number of patients to receive the beneficial treatment of the medical staff. It was, however, necessary sometimes to have a watch over what was done. For himself he wanted to know what became of his money, and to watch how the patients were treated by the doctors, and the secretary was the very man to go between the medical men and the Governers.

The Rev. W. E. Haigh appealed to the physicians—to

* *Vide* The Hospital, Vol. IV., p. 334, August 25, 1888.

whom they were all indebted and who were all respected—that they should act generously and hold out an open hand to Mr. Austin, so that the very unfortunate dispute might be settled in a friendly and peaceful manner (hear, hear).

The Rev. Mr. Webster also earnestly appealed to the medical staff and the secretary to effect a reconciliation. He proposed as an amendment, "That this meeting hopes that a reconciliation may at once take place between the medical staff and the late secretary."

Mr. Taylor suggested another amendment as follows, which Mr. Webster seconded and accepted in place of his own: "That the council be respectfully requested to reconsider the resignation of the secretary, the subscribers being of opinion that the action taken by the secretary in bringing questions of medical policy before the council was done in good faith and in the best interests of the institution."

Mr. Burdett, who was received with interruptions, said he had recently qualified as a governor to enable him to understand all the facts. He pointed out that the passing of either amendment, as matters stood, could not advance matters nor lead to a settlement, but rather the contrary. Certain statements had been made by the secretary reflecting vitally on the good faith, skill, and character of the medical staff, which statements the council on investigation declared to be unfounded and unworthy. Yet the secretary, Mr. Austin, had in effect reiterated and emphasised these statements in their hearing at that meeting. Now how could anyone hope for a reconciliation unless or until Mr. Austin again ascended the platform, and there and then withdrew his statements and apologised for his indiscretion and conduct? There must be order and good government in a hospital, as elsewhere, and if the late secretary did not take this course now, no independent governor could vote for the amendment, which must fail in its object. He was in favour of peace, because he could not forget the many poor people suffering from consumption in this vast city who had far too few beds already for their needs. Still, as matters stood, unless the secretary apologised and withdrew there and then, it would be impossible for him to again serve as secretary of that hospital.

The tone of the meeting now changed, and several of the governors tried to throw oil upon the waters. This line of action, however, did not, in the attitude assumed by Mr. Austin, commend itself to the chairman, who took upon himself the whole responsibility of what had been done by the council. If, as Mr. Austin had said, the medical gentlemen had been guilty of carrying out scientific experiments upon patients and of misapplying the uses of the hospital, why had he not informed the council earlier? I ask you as governors (continued the chairman), why did he not ever bring that under the notice of the council if it was such a grave matter? This has been going on for three years, why did he not bring it forward at the last annual meeting? ("Oh, oh," and a voice: "You are acting unfairly"). The Chairman, continuing, said that no one could be more ready than himself to acknowledge the interest which Mr. Austin had taken in the hospital. But it was a serious matter to suggest, as had been done, that the staff had been guilty of carrying on experiments (hear, hear). That which he (the chairman) had communicated of Mr. Austin's was not at all a privileged communication. There was no doubt that the hospital was essentially a poor man's and poor woman's hospital, and in the action he (Lord Charles Bruce) was taking he was thinking of his poor suffering brothers and sisters (hear, hear, and cries of "Vote.")

The amendment of Mr. Taylor, upon being submitted to the vote, was, however, supported by fifty-seven governors, whereas twenty-two only voted against it. Upon being carried as a substantive motion the long proceedings closed with a vote of thanks to the chairman.

